# Supplementary material for: The Dimeric Form of HPV16 E6 Is Crucial to Drive YAP/TAZ Upregulation through the Targeting of hScrib
Source: Cancers (Basel). 2021 Aug 13;13(16):4083. doi: 10.3390/cancers13164083 (PMC8393709; doi:10.3390/cancers13164083)
Supplement: Supplementary file 1 [file cancers-13-04083-s001.zip › Supplementary Figures.pdf]

cancers

MDPI

# The Dimeric Form of HPV16 E6 Is Crucial to Drive YAP/TAZ Upregulation through the Targeting of hScrib

**Lorenzo Messa, Marta Celegato, Chiara Bertagnin, Beatrice Mercorelli, Gualtiero Alvisi, Lawrence Banks, Giorgio Palù and Arianna Loregian**

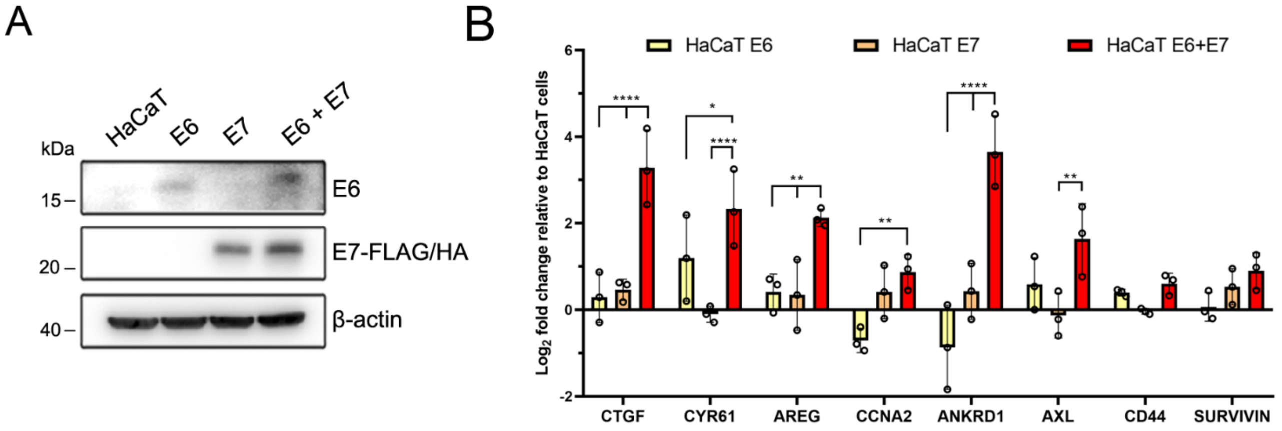


**Figure S1.** Expression control of the cell lines used in the experiments assessing the requirement of both E6 and E7 to promote YAP/TAZ signaling and stemness properties. (**A**) Western blot analysis of transiently transfected HaCaT cells transfected with empty vector or overexpressing either HPV16 E6, HPV16 E7 or both, used for qPCR and 3D spheroid experiments shown in Figure 1A and 1B. β-actin was used as a loading control. (**B**) Non-normalized log2 fold-change values of YAP/TAZ-target genes expression in confluent HaCaT cells overexpressing either HPV16 E6, HPV16 E7 or both versus confluent parental empty vector-transfected HaCaT cells. Data are Mean ± SD of three independent experiments performed in triplicate. * $p < 0.05$, ** $p < 0.01$, **** $p < 0.0001$ determined with two-way ANOVA with Dunnett's multiple-comparisons test.

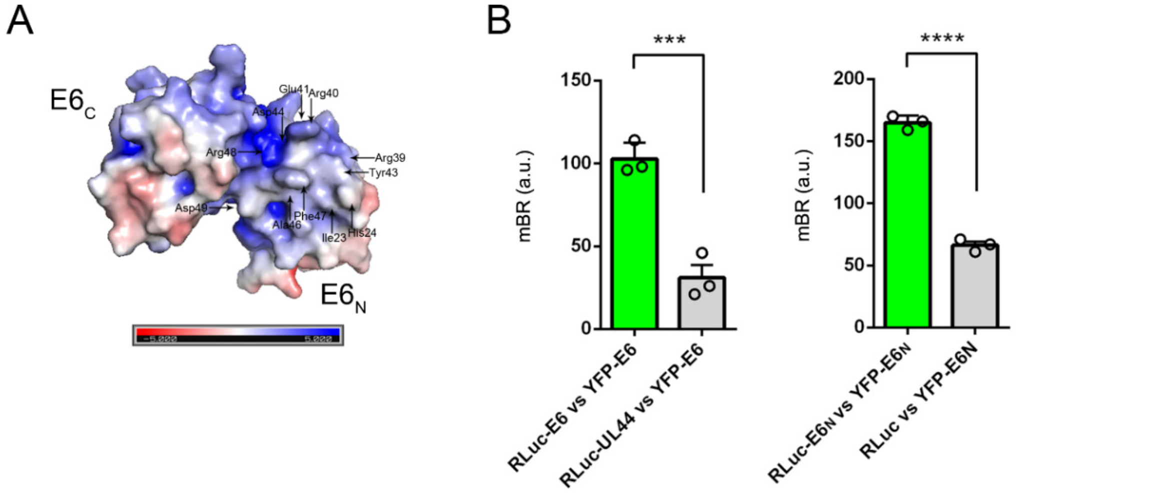


**Figure S2.** The hydrophobic N-terminal $\alpha$2 helix of HPV16 E6 mediates E6 homodimerization. (**A**) Electrostatic surface representation of E6 with surface-exposed $\alpha$2 helix residues indicated, showing the general hydrophobic nature of the binding region. Red: negatively charged surfaces; blue: positively charged surfaces; white: neutral surfaces. $E6_N$ and $E6_C$: N- and C-terminal domains. Model created with APBS tool in PyMOL (PDB code: 4xr8). (**B**) Comparison of E6 self-association signals with negative controls measured through BRET assays in HEK 293T cells expressing full-length E6 proteins (left graph) or N-terminal fragments (right graph). Negative controls represent the interaction signals of YFP-E6 or YFP-$E6_N$ with two unrelated proteins (RLuc-tagged UL44 from human cytomegalovirus or RLuc, respectively). Data are Mean ± SD of three independent experiments performed in triplicate. *** $p < 0.001$, **** $p < 0.0001$ determined with unpaired two-tailed $t$ tests.

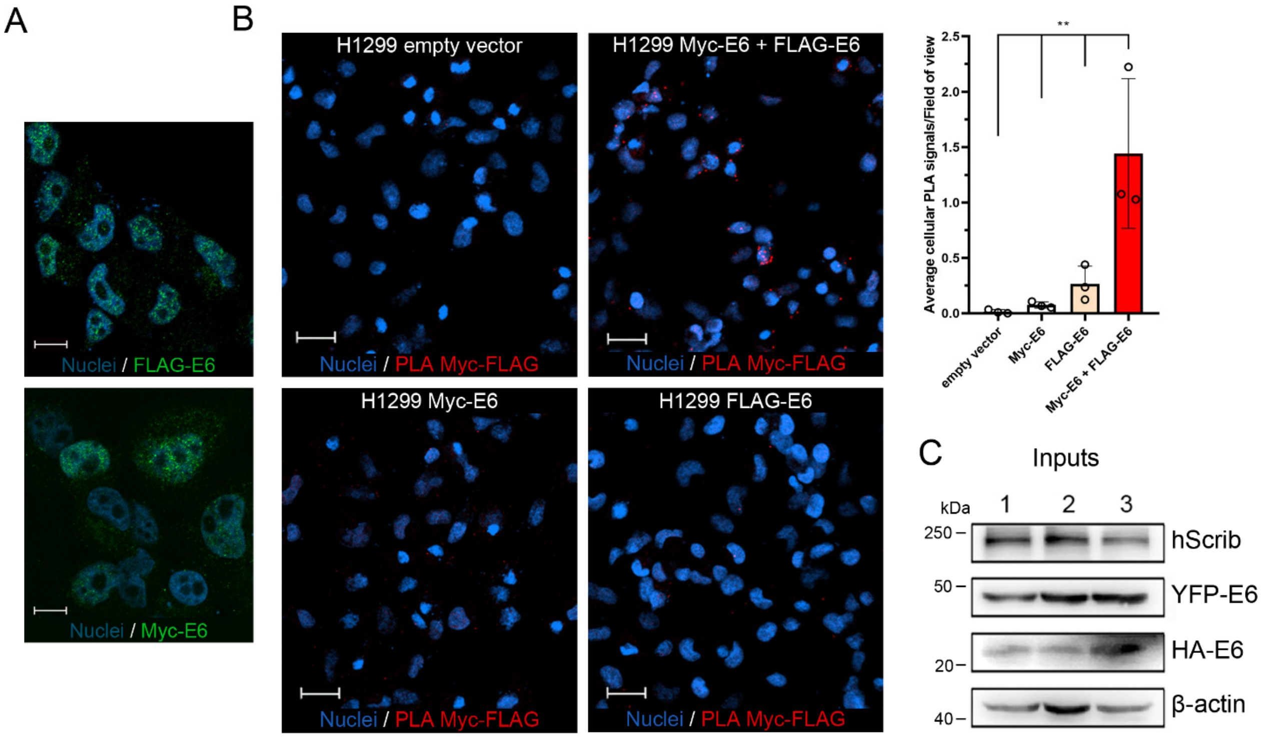


**Figure S3.** Controls of the samples used in the experiments assessing E6 homodimerization in the cytoplasm and its link to the E6/hScrib interaction. (**A**) Representative immunofluorescence images of H1299 cells expressing FLAG-E6 (green fluorescence, upper image) or Myc-E6 (green fluorescence, lower image) used for Proximity Ligation Assays. Scale bars: 10 µm. (**B**) Representative confocal images of Myc-FLAG Proximity Ligation Assays (PLA) in H1299 cells. Negative controls represent Myc-FLAG PLA in empty vector-expressing cells or in cells overexpressing only Myc-E6 or FLAG-E6. Right graph shows the quantification of PLA signals normalized over the number of cells present in three different fields of view for each sample, with $n_{H1299\text{-empty vector}} = 361$, $n_{H1299\text{-Myc-E6}} = 227$, $n_{H1299\text{-FLAG-E6}} = 450$ and $n_{H1299\text{-Myc-E6 + FLAG-E6}} = 324$. Scale bars: 25 µm. ** $p < 0.01$ determined with one-way ANOVA with Dunnett's multiple-comparisons test. (**C**) Western blot analysis for YFP-tag, HA-tag, and endogenous hScrib of H1299 cell lysates expressing YFP-tagged and HA-tagged E6 proteins used for hScrib immunoprecipitations in Figure 2C. β-actin was used as a loading control. In (**A**) and (**B**) nuclei were stained with DRAQ5.

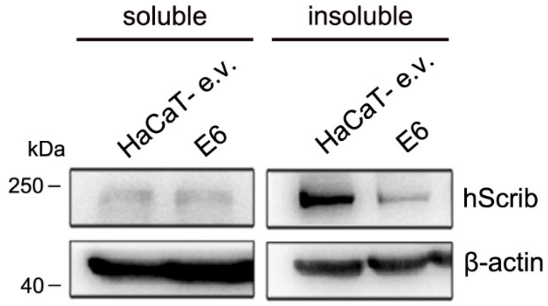


**Figure S4.** HPV16 E6 targets the insoluble fraction of hScrib. Western blot analysis of the soluble or insoluble fractions of HaCaT-e.v. and HaCaT-E6 cell lysates showing the dramatic reduction of the insoluble fraction of endogenous hScrib following E6 overexpression. Cells were cultivated at complete confluence before harvesting. β-actin was used as a loading control.

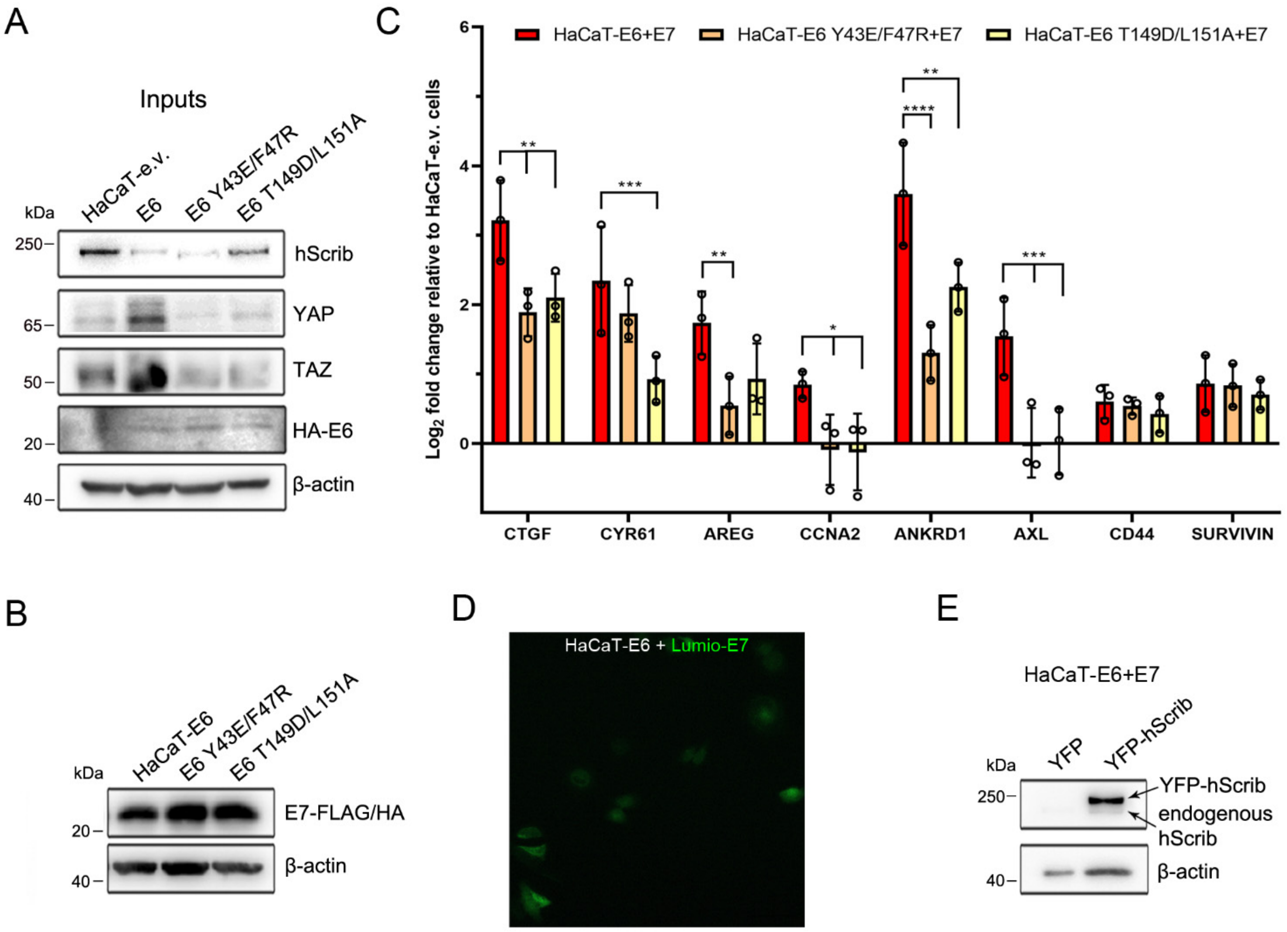


**Figure S5.** Controls of the experimental procedures assessing the contribution of hScrib targeting mediated by dimeric E6 to promote YAP/TAZ signaling. (**A**) Western blot analysis of endogenous hScrib, YAP and TAZ in stable HaCaT cell lines overexpressing HA-tagged E6 proteins or bearing empty vector used for hScrib immunoprecipitations in Figure 5A. β-actin was used as a loading control. (**B**) Western blot analysis of HaCaT-E6, HaCaT-E6 Y43E/F47R, and HaCaT-E6 T149D/L151A cells overexpressing HPV16 E7-FLAG/HA used for YAP/TAZ-target genes expression analysis shown in Figure 5C. β-actin was used as a loading control. (**C**) Non-normalized log2 fold-change values of YAP/TAZ-target genes expression in confluent HaCaT-E6, HaCaT-E6 Y43E/F47R and HaCaT-E6 T149D/L151A cells overexpressing HPV16 E7-FLAG/HA versus confluent HaCaT-e.v. cells. Data are Mean ± SD of three independent experiments performed in triplicate. * $p < 0.05$, ** $p < 0.01$, *** $p < 0.001$, **** $p < 0.0001$ determined with two-way ANOVA with Dunnett's multiple-comparisons test. (**D**) Representative images of HaCaT-E6 cells stably overexpressing wild-type Lumio-tagged HPV16 E7 (green fluorescence). Scale bars: 50 μm. (**E**) Western blot analysis of endogenous hScrib and exogenous overexpressed YFP-hScrib in HaCaT-E6+E7 cells used for the generation of spheroids shown in Figure 5E. β-actin was used as a loading control.

**Table S1.** Antibodies used in this study.

| Antibody (clone–application–dilution) | Source | Identifier |
|---|---|---|
| Mouse anti-p53 (DO-1) (WB 1:4000) | Santa Cruz | Cat# sc-126 |
| Goat anti-HPV16 E6 (N-17) (WB 1:200) | Santa Cruz | Cat# sc-1584 |
| Mouse anti-HPV16/18 E6 (C1P5) (WB 1:100) | Santa Cruz | Cat# sc-460 |
| Mouse anti-HPV16 E7 (NM2) (WB 1:500) | Santa Cruz | Cat# sc-65711 |
| Mouse anti-HA (F-7) (WB 1:500) | Santa Cruz | Cat# sc-739 |
| Rabbit anti-HA (Y-11) (WB 1:500) | Santa Cruz | Cat# sc-805 |
| Goat anti-hScrib (C-20) (IF 1:1000) | Santa Cruz | Cat# sc-11049 |
| Mouse anti-hScrib (D-2) (IP) | Santa Cruz | Cat# sc-374139 |
| Mouse anti-YAP (63.7) (WB 1:1000) | Santa Cruz | Cat# sc-101199 |
| Mouse anti-β-actin (WB 1:8000) | Sigma-Aldrich | Cat# A5441 |
| Rabbit anti-hScrib (WB 1:4,000 / IF 1:500) | Sigma-Aldrich | Cat# HPA064312 |
| Goat anti-YAP (WB 1:1000) | Sigma-Aldrich | Cat# SAB2501938 |
| Rabbit anti-TAZ (WB 1:1000) | Sigma-Aldrich | Cat# HPA007415 |
| Rabbit anti-FLAG (IF/PLA 1:200) | Sigma-Aldrich | Cat# F7425 |
| Mouse anti-Myc (9E10) (IF/PLA 1:1000) | Sigma-Aldrich | Cat# M4439 |
| Rabbit anti-hScrib (IP) | Invitrogen | Cat# PA5-28628 |
| Mouse anti-GFP (3F8.2) (WB 1:1000) | Millipore | Cat# MAB1083 |
| Goat anti-rabbit IgG-HRP (WB 1:4000) | Millipore | Cat# 12-348 |
| Goat anti-mouse IgG-HRP (WB 1:4000) | Millipore | Cat# 12-349 |
| Rabbit anti-goat IgG-HRP (WB 1:4000) | Millipore | Cat# AP106P |
| Donkey anti-mouse IgG-Alexa 488 (IF 1:1000) | Invitrogen | Cat# A21202 |
| Donkey anti-rabbit IgG-Alexa 488 (IF 1:1000) | Invitrogen | Cat# A21206 |
| Donkey anti-rabbit IgG-Alexa 555 (IF 1:1000) | Invitrogen | Cat# A31572 |
| Donkey anti-goat IgG-Alexa 555 (IF 1:1000) | Invitrogen | Cat# A21432 |

WB: Western blotting; IF: Immunofluorescence; IP: Immunoprecipitation; PLA: Proximity Ligation assay.

**Table S2.** Oligonucleotides used in this study for qPCR experiments.

| Oligonucleotide | Source[a] |
|---|---|
| GAPDH qPCR forward primer<br>5′-TCATTTCCTGGTATGACAACG-3′ | Enge et al., 2009 |
| GAPDH qPCR reverse primer<br>5′-ATGTGGGCCATGAGGT-3′ | Enge et al., 2009 |
| CTGF qPCR forward primer<br>5′-AGGAGTGGGTGTGTGACGA-3′ | Zanconato et al., 2015 |
| CTGF qPCR reverse primer<br>5′-CCAGGCAGTTGGCTCTAATC-3′ | Zanconato et al., 2015 |
| CYR61 qPCR forward primer<br>5′-CCTTGTGGACAGCCAGTGTA-3′ | This paper |
| CYR61 qPCR reverse primer<br>5′-ACTTGGGCCGGTATTTCTTC-3′ | This paper |
| AREG qPCR forward primer<br>5′-CGAACCACAAATACCTGGCTA-3′ | He et al., 2015 |
| AREG qPCR reverse primer<br>5′-TCCATTTTTGCCTCCCTTTT-3′ | He et al., 2015 |
| CCNA2 qPCR forward primer<br>5′-AGTAAACAGCCTGCGTTCACC-3′ | Amatori et al., 2017 |
| CCNA2 qPCR reverse primer<br>5′-GAGGGACCAATGGTTTTCTGG-3′ | Amatori et al., 2017 |
| ANKRD1 qPCR forward primer<br>5′-AGTAGAGGAACTGGTCACGG-3′ | Chang et al., 2015 |
| ANKRD1 qPCR reverse primer<br>5′-TGTTTCTCGCTTTTCCACTGTT-3′ | Chang et al., 2015 |
| AXL qPCR forward primer<br>5′-CCAGGACACCCCAGAGGTGCTAAT-3′ | Uribe et al., 2017 |
| AXL qPCR reverse primer<br>5′-TGGTGGACTGGCTGTGCTTGC-3′ | Uribe et al., 2017 |
| CD44 qPCR forward primer<br>5′-TGGCATCCCTCTTGGCCTTGG-3′ | Johansson et al., 2017 |
| CD44 qPCR reverse primer<br>5′-TGAGACTTGCTGGCCTCTCCGT-3′ | Johansson et al., 2017 |
| SURVIVIN qPCR forward primer<br>5′-GACCACCGCATCTCTACATTC-3′ | Vargas and Vivas-Mejìa, 2013 |
| SURVIVIN qPCR reverse primer<br>5′-TGCTTTTTATGTTCCTCTATGGG-3′ | Vargas and Vivas-Mejìa, 2013 |

## References


1. Amatori, S.; Persico, G.; Fanelli, M. Real-time quantitative PCR array to study drug-induced changes of gene expression in tumor cell lines. *J. Cancer Metastasis Treat* **2017**, *3*, 90–99.
2. Chang, A.T.; Liu, Y.; Ayyanathan, K.; Benner, C.; Jiang, Y.; Prokop, J.W.; et al. An evolutionarily conserved DNA architecture determines target specificity of the TWIST family bHLH transcription factors. *Genes. Dev.* **2015**, *29*, 603–616.
3. Enge, M.; Bao, W.; Hedström, E.; Jackson, S.P.; Moumen, A.; Selivanova, G. MDM2-dependent downregulation of p21 and hnRNP K provides a switch between apoptosis and growth arrest induced by pharmacologically activated p53. *Cancer Cell.* **2009**, *15*, 171–183.
4. He, C.; Mao, D.; Hua, G.; Lv, X.; Chen, X.; Angeletti, P.C.; et al. The Hippo/YAP pathway interacts with EGFR signaling and HPV oncoproteins to regulate cervical cancer progression. *EMBO Mol. Med.* **2015**, **7**, 1426–1449.
5. Johansson, E.; Grassi, E.S.; Pantazopoulou, V.; Tong, B.; Lindgren, D.; Berg, T.J.; et al. CD44 Interacts with HIF-2$\alpha$ to Modulate the Hypoxic Phenotype of Perinecrotic and Perivascular Glioma Cells. *Cell Rep.* **2017**, *20*, 1641–1653.
6. Uribe, D.J.; Mandell, E.K.; Watson, A.; Martinez, J.D.; Leighton, J.A.; Ghosh, S.; Rothlin, C.V. The receptor tyrosine kinase AXL promotes migration and invasion in colorectal cancer. *PLoS One* **2017**, *12*, e0179979.
7. Vargas, I.M.E.; Vivas-Mejía, P.E. Assessment of mRNA splice variants by qRT-PCR. *Methods Mol. Biol.* **2013**, *1049*, 171–186.

8. Zanconato, F.; Forcato, M.; Battilana, G.; Azzolin, L.; Quaranta, E.; Bodega, B.; et al. Genome-wide association between YAP/TAZ/TEAD and AP-1 at enhancers drives oncogenic growth. *Nat. Cell Biol.* **2015**, *17*, 1218–1227.
