# Supplementary material for: The Dimeric Form of HPV16 E6 Is Crucial to Drive YAP/TAZ Upregulation through the Targeting of hScrib
Source: Cancers (Basel). 2021 Aug 13;13(16):4083. doi: 10.3390/cancers13164083 (PMC8393709; doi:10.3390/cancers13164083)
Supplement: Supplementary file 1 [file cancers-13-04083-s001.zip › Supplementary Information.pdf]

Supplementary Information

Original uncropped blots displayed in Figure 2A for HEKn cells. βactins shown in the figure are those corresponding to the blot of TAZ. YAP was probed through a second run and incubation with a YAP-specific antibody.

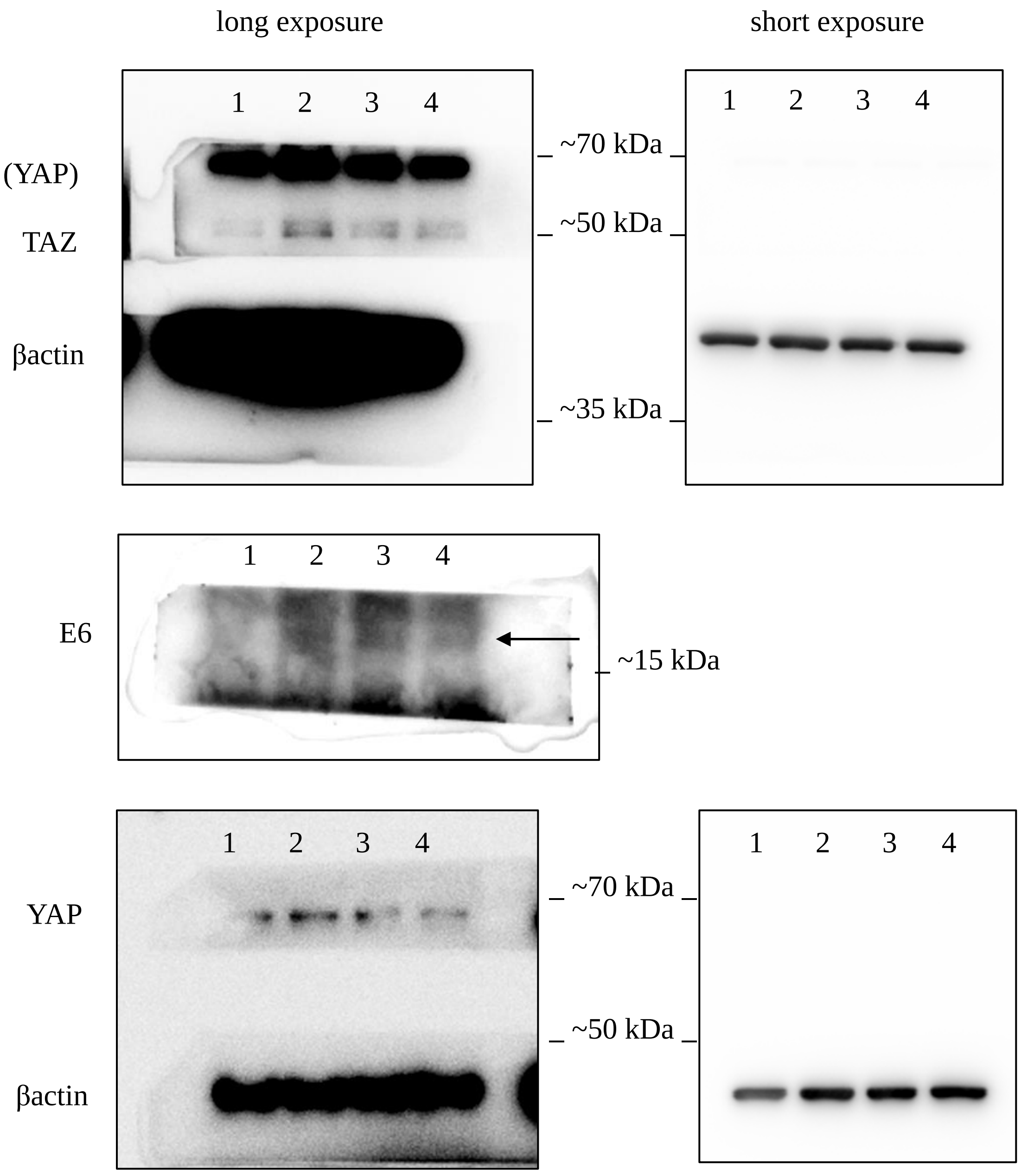

1: empty vector; 2: E6; 3: E6 Y43E/F47R; 4: E6 T149D/L151A

Original uncropped blots displayed in Figure 2A for C33A cells. YAP was reprobed on the same membrane after stripping, cutting and reprobing with a YAP-specific antibody.

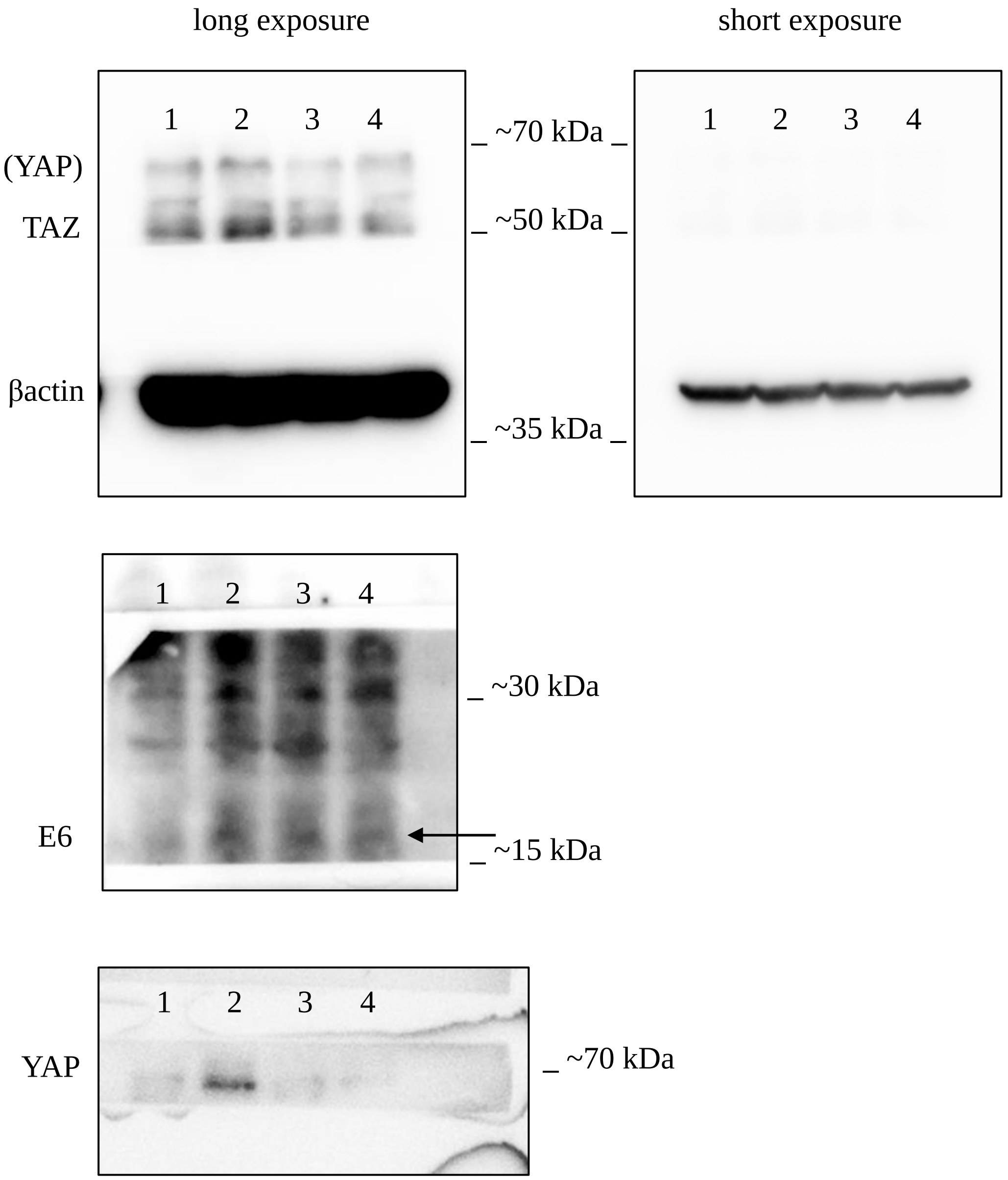

1: empty vector; 2: E6; 3: E6 Y43E/F47R; 4: E6 T149D/L151A

Original uncropped blots displayed in Figure 2A for H1299 cells. βactins shown in the figure are those corresponding to the blot of TAZ. YAP was probed through a second run and incubation with a YAP-specific antibody.

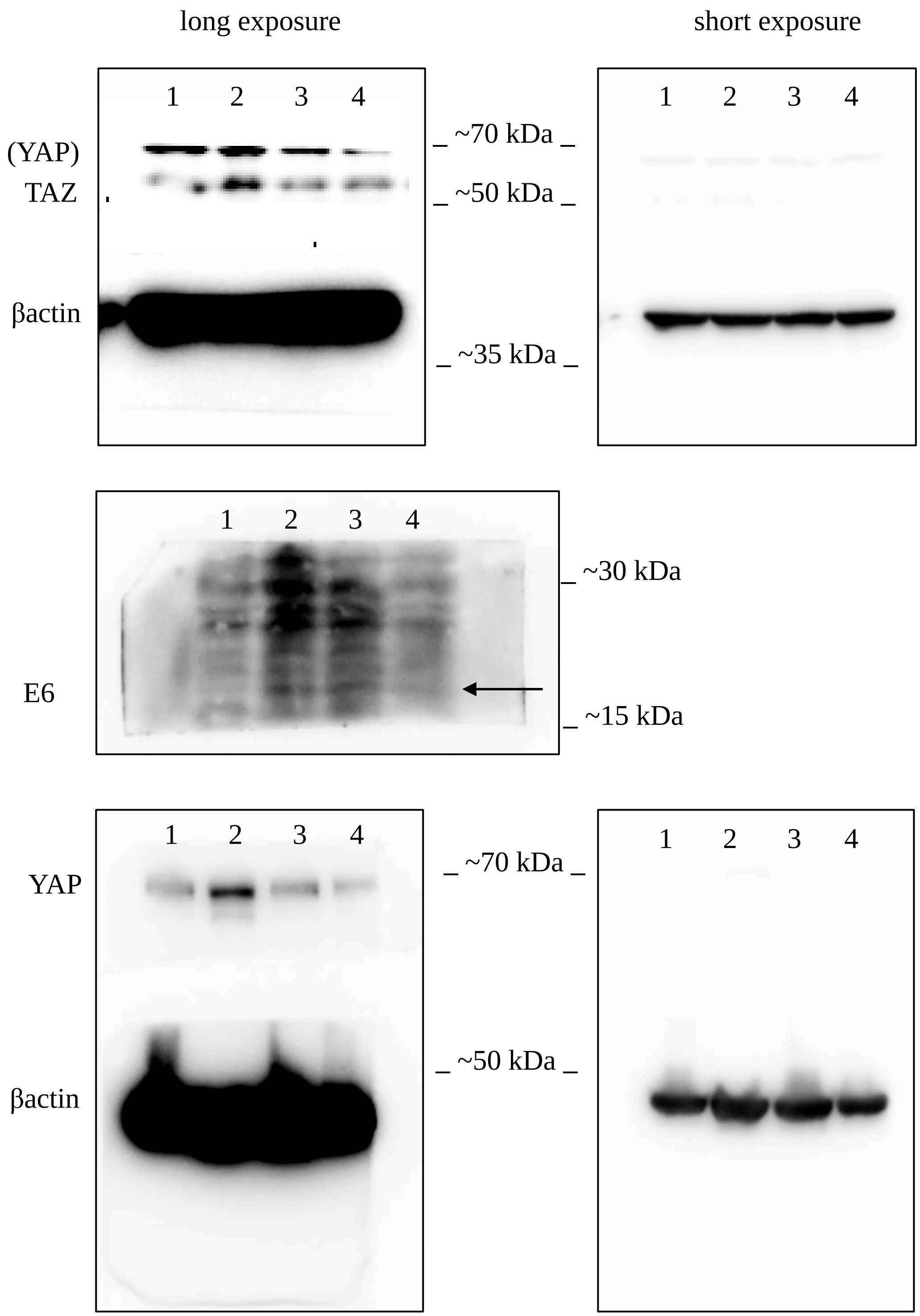

1: empty vector; 2: E6; 3: E6Y43E/F47R; 4: E6 T149D/L151A

Original uncropped blots displayed in Figure 2E. βactins were blotted onto the same membrane wherein p53 was visualized after stripping and reprobing.

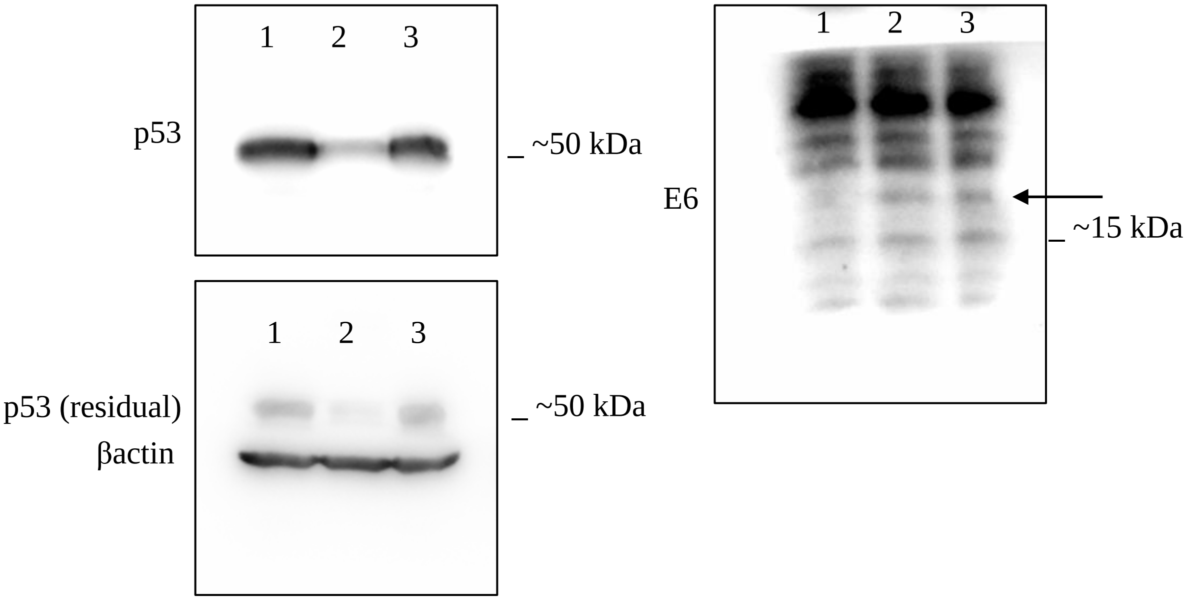

1: 293T empty vector
2: 293T E6
3: 293T E6 Y43E/F47R

Original uncropped blots displayed in Figure 3C. All proteins were blotted on the same membrane.

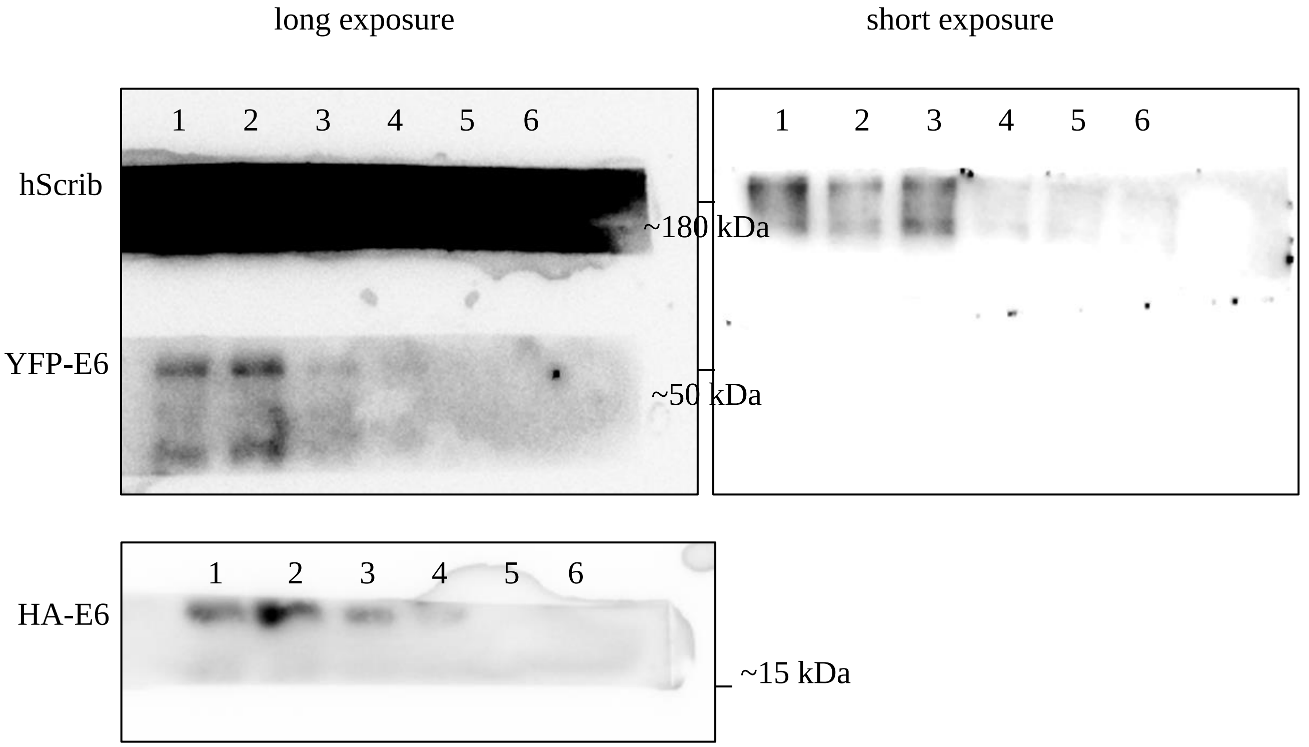

1: H1299 IP-hScrib YFP-E6 + HA-E6
2: H1299 IP-hScrib YFP-E6 T149D/L151A + HA-E6
3: H1299 IP-hScrib YFP-E6 T149D/L151A + HA-E6 Y43E/F47R
4: H1299 Control-IP YFP-E6 + HA-E6
5: H1299 Control-IP YFP-E6 T149D/L151A + HA-E6
6: H1299 Control-IP YFP-E6 T149D/L151A + HA-E6 Y43E/F47R

Original uncropped blots displayed in Figure 3D. Proteins were blotted on the same membrane.

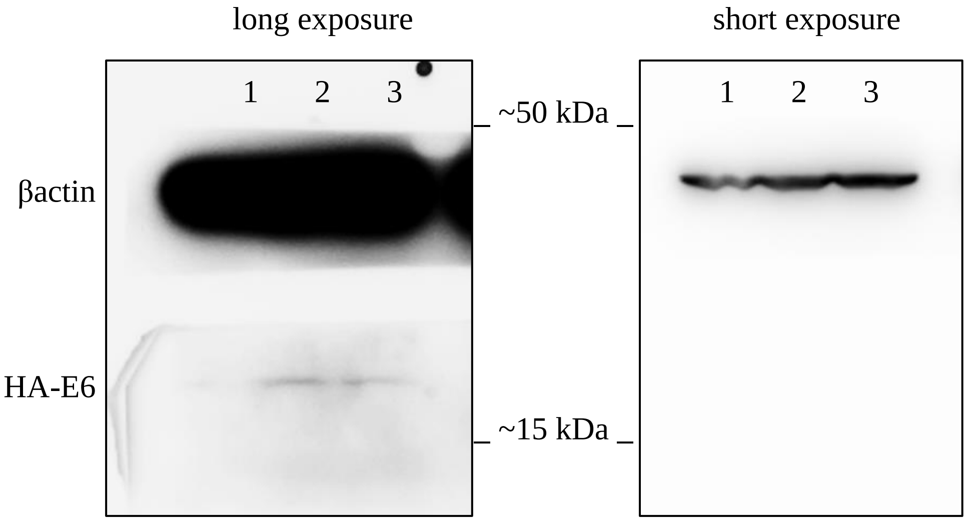

1: 293T YFP-hScrib + Rluc-E6 T149D/L151A + empty vector
2: 293T YFP-hScrib + Rluc-E6 T149D/L151A + HA-E6
3: 293T YFP-hScrib + Rluc-E6 T149D/L151A + HA-E6 Y43E/F47R

Original uncropped blots displayed in Figure 5A. All proteins were blotted on the same membrane.

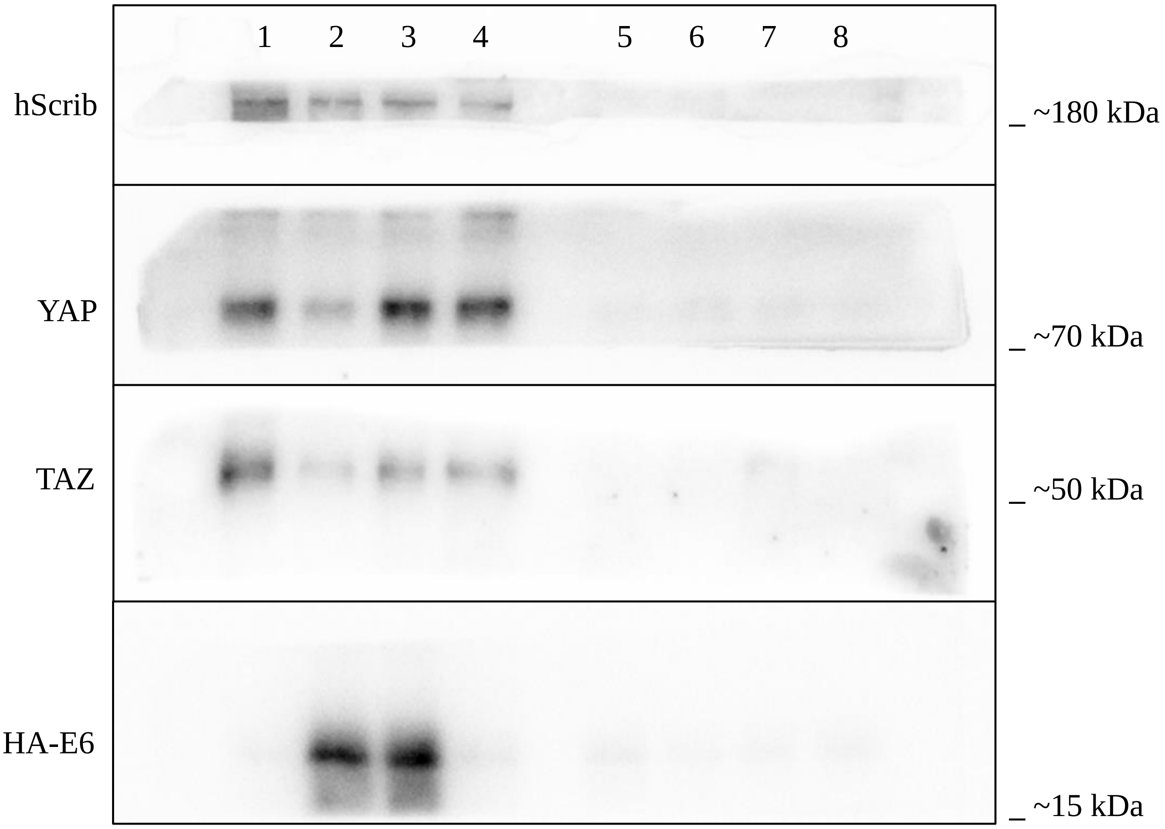

1: IP-hScrib HaCaT-empty vector
2: IP-hScrib HaCaT-E6
3: IP-hScrib HaCaT-E6 Y43E/F47R
4: IP-hScrib HaCaT-E6 T149D/L151A;
5: Control-IP HaCaT-empty vector
6: Control-IP HaCaT-E6
7: Control-IP HaCaT-E6 Y43E/F47R
8: Control-IP HaCaT-E6 T149D/L151A

Original uncropped blots displayed in Figure S1. Proteins were blotted on the same membrane.

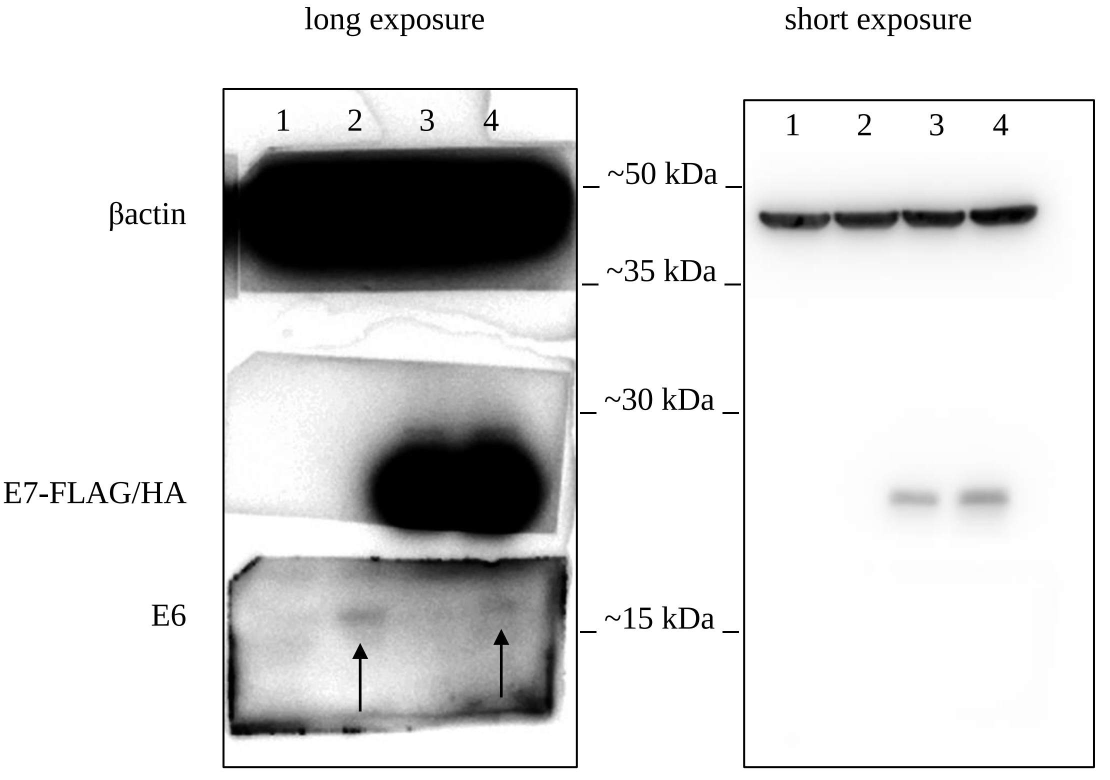

1: HaCaT empty vector
2: HaCaT E6
3: HaCaT E7-FLAG/HA
4: HaCaT E6 + E7-FLAG/HA

Original uncropped blots displayed in Figure S3C. Proteins were blotted on the same membrane. βactins were blotted after stripping and reprobing onto the same slice wherein YFP-E6 was visualized.

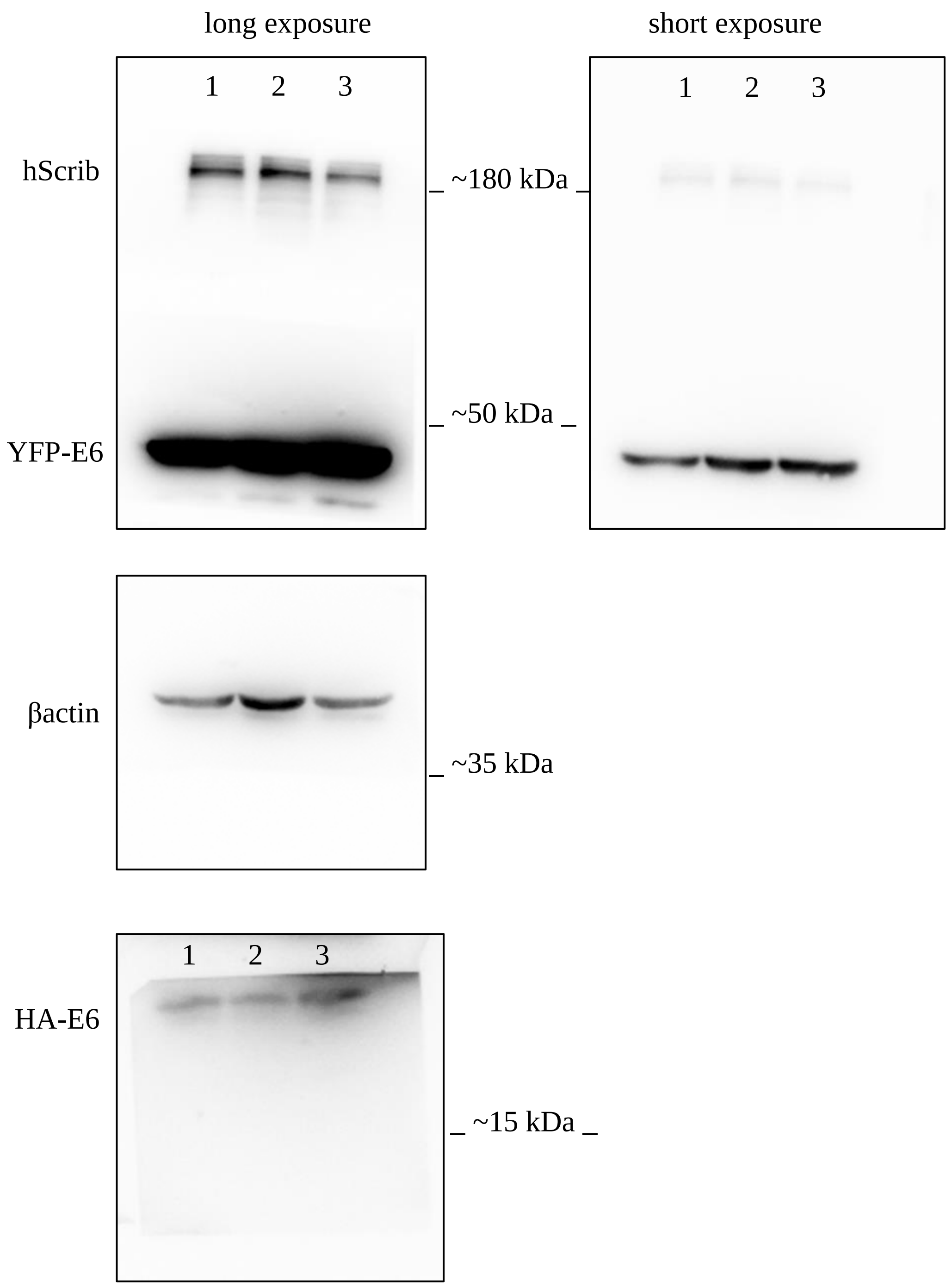

1: H1299 YFP-E6 + HA-E6
2: H1299 YFP-E6 T149D/L151A + HA-E6
3: H1299 YFP-E6 T149D/L151A + HA-E6 Y43E/F47R

Original uncropped blots displayed in Figure S4 for soluble extracts. Proteins were blotted on the same membrane. Middle slice show a protein blot not related to the study.

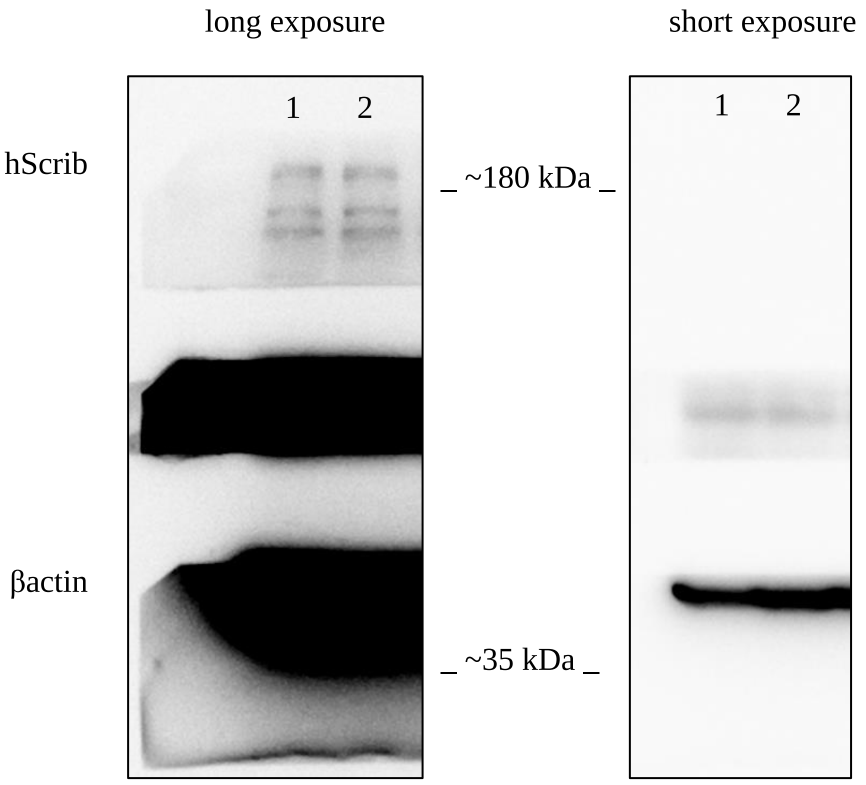

Original uncropped blots displayed in Figure S4 for insoluble extracts. Proteins were blotted on the same membrane.

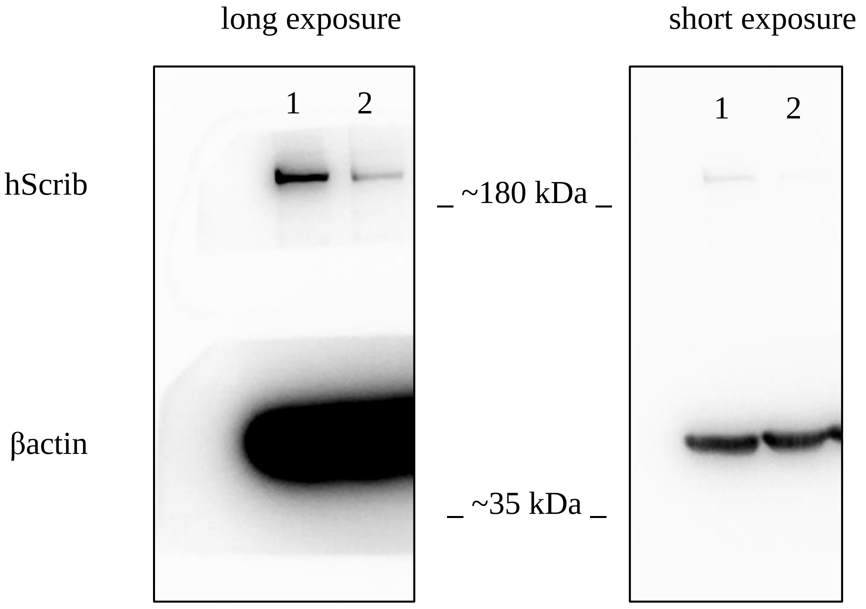

1: HaCaT-empty vector; 2: HaCaT-E6

Original uncropped blots displayed in Figure S5A. βactins shown in the figure are those corresponding to the blot of TAZ.

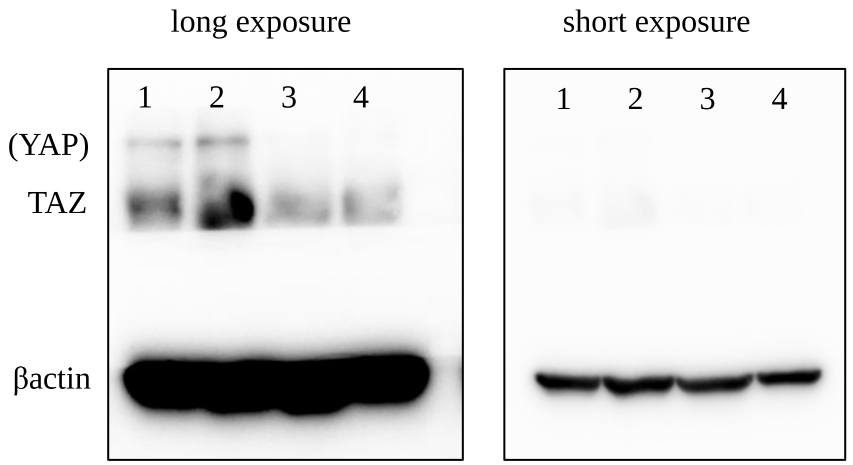

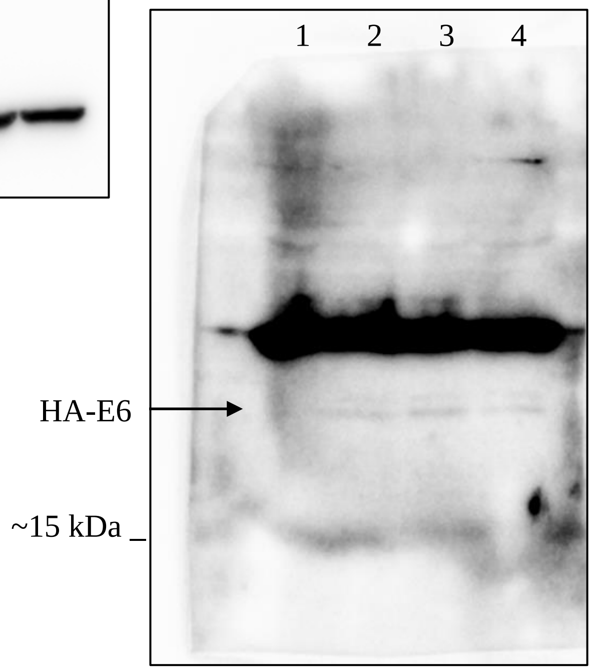

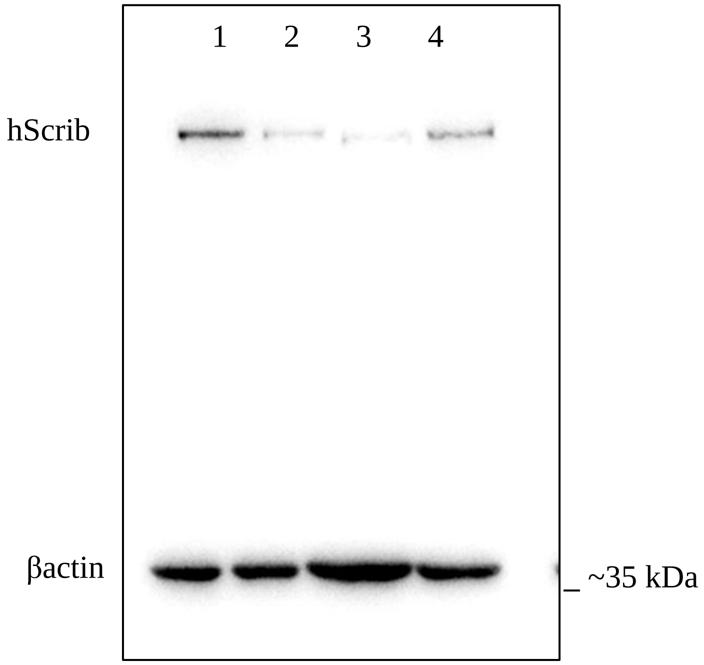

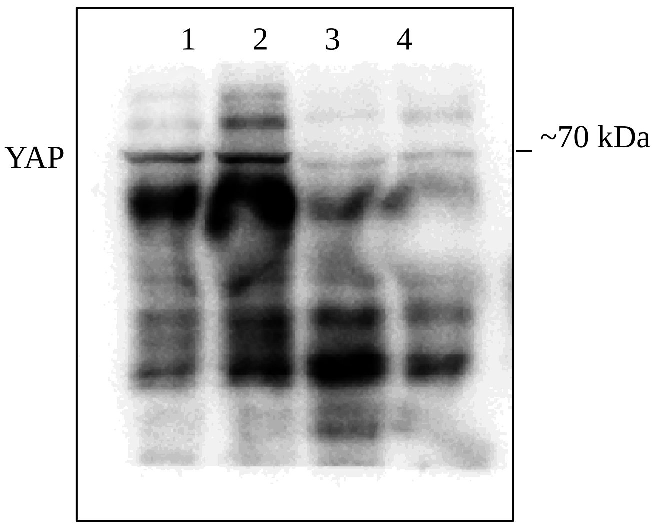

1: HaCaT-empty vector
2: HaCaT-E6
3: HaCaT-E6 Y43E/F47R
4: HaCaT-E6 T149D/L151A

Original uncropped blots displayed in Figure S5D. Proteins were blotted on the same membrane.

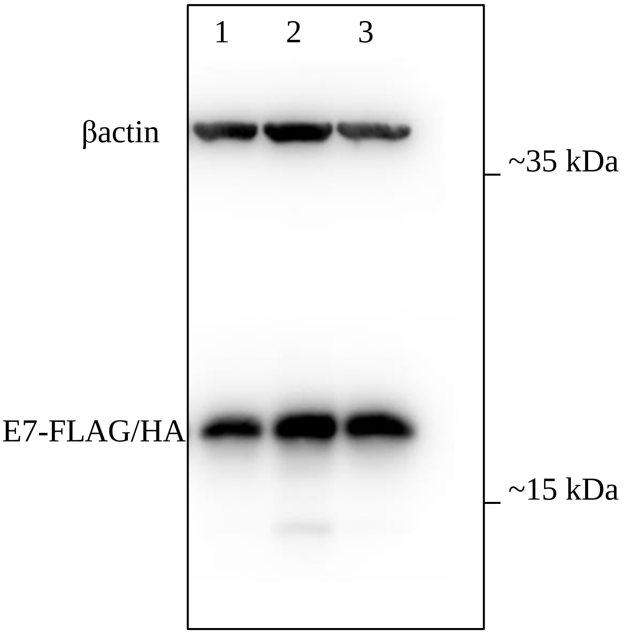

1: HaCaT-E6 + E7-FLAG/HA
2: HaCaT-E6 Y43E/F47R + E7-FLAG/HA
3: HaCaT-E6 T149D/L151A + E7-FLAG/HA

Original uncropped blots displayed in Figure S5F. Proteins were blotted on the same membrane. Middle slice show a protein blot not related to the study.

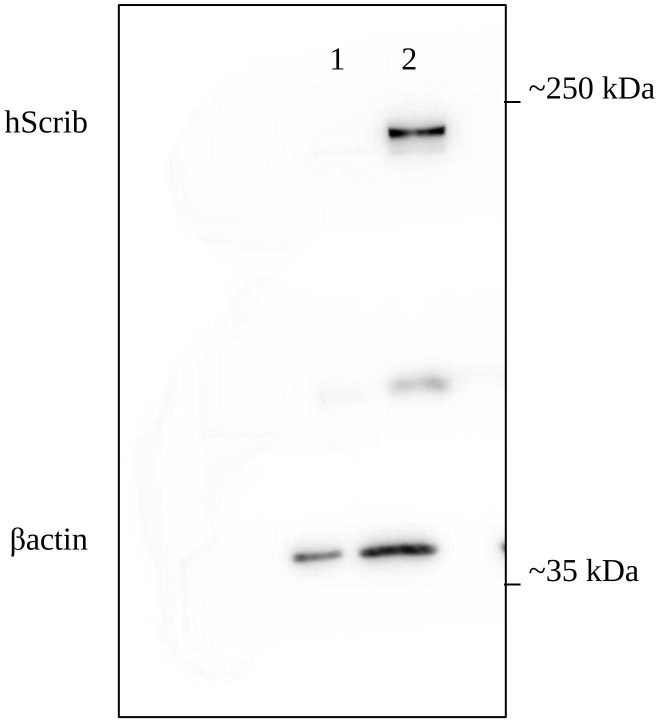

1: HaCaT-E6+E7 YFP
2: HaCaT-E6+E7 YFP-hScrib
